# On the Brink: How Biology and Humans Affect Extinction Risk

**DOI:** 10.1371/journal.pbio.0020208

**Published:** 2004-07-13

Close to a quarter of the world's mammals are at high risk of extinction. Save for the periodic “great extinctions,” mammalian extinction has been a relatively rare event in geological terms, with one species disappearing from the fossil record every 1,000 years or so. Over the past 400 years, species have been disappearing 50 times faster than this “background” rate, with one vanishing every sixteen years. Human population growth and all its consequences—habitat destruction, propagation of invasive species, poaching—are largely to blame. Top predators often suffer heavily from encounters with humans, especially when those predators are perceived as economic threats. Thirty-four Mexican gray wolves have been reintroduced in Arizona since 1998, and five have been shot, reportedly by ranchers.[Fig pbio-0020208-g001]

**Figure pbio-0020208-g001:**
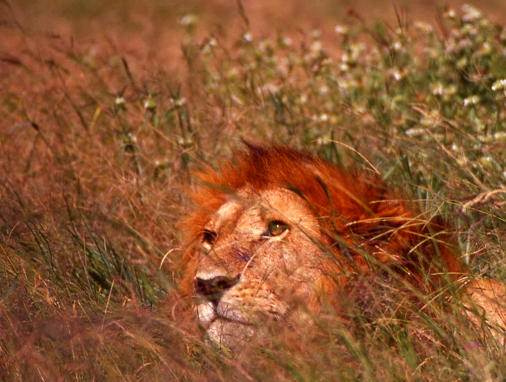
The IUCN lists lions as vulnerable. Photo, with permission, by Nicky Jenner, Institute of Zoology, London

Species in the most densely populated areas are expected to face the greatest risk, yet some survive while others perish, suggesting biological factors play a role in their fate. If, for example, the same external force drastically reduces populations of species with different biological profiles, then a species with a relatively short gestation period may stand a better chance of recovering than a long-gestating species.

Effective conservation strategies depend on understanding which factors are likely to increase extinction risk, but it's unclear how important intrinsic biological traits are relative to external pressures from humans and whether biology's influence on survival depends on the intensity of the threat. Ecologists often use human population density as a proxy for anthropogenic threats such as habitat destruction and hunting. To tease out the relative importance of all these factors, Marcel Cardillo et al. analyzed the impact of various biological traits and human population density on extinction risk in the mammal order Carnivora, which includes the red panda, lion, and members of the photogenic weasel-like viverrid family. By identifying the most salient factors in predicting extinction, the authors have created a model to identify those species at greatest risk.

The biology of a species combined with human population density, the researchers found, is a stronger predictor of risk than exposure to humans alone; those biological traits that increase risk vary depending on a species' exposure to human populations. Carnivores with low exposure to humans, for example, are likely to be at greater risk if their population density is low and they have small ranges, possibly because this makes them more vulnerable to loss of habitat. Species living near densely populated human areas must often contend with hunting and other direct threats on top of habitat loss and are more at risk if they also have long gestation periods—they can't repopulate fast enough to offset the additional pressures. Based on projected human population growth, this model predicts the addition of a number of species—mostly from Africa, where population growth rates largely exceed the global average—to the endangered list by the year 2030. Most of these species—including African viverrids such as the common genet, which not only lives in areas where human populations are rapidly expanding but is also biologically predisposed to decline—are currently considered a low conservation priority.

While it's possible that the direct effects of human population density are past—that is, species most sensitive to human incursions are already gone—human population density likely modulates biology. That might explain why gestation length didn't predict risk for species living in sparsely populated areas—all else being equal, their numbers remained relatively stable. A species with a small population forebodes a high extinction risk regardless of human density, though species with long gestation periods, again, face greater danger in the company of humans.

Altogether, these results suggest that as human population pressures increase, the importance of species-specific biology in predicting extinction risk also increases, with biology affecting which species are most vulnerable to external threats. With most conservation efforts focused on damage control, these findings make the case for interceding *before* a species reaches the brink of extinction. “There is no room for complacency about the security of species,” the authors warn, “simply because they are not currently considered threatened.”

